# Boston Meeting

**Published:** 1857-12

**Authors:** 


### BOSTON MEETING.

MESSRS. EDITORS,—*Gentlemen*:—I see, by the Dental periodicals, as well as by the newspapers, that the profession of which I claim to be an humble member, has had another jubilant convocation, and has not only stirred up the Yankees away down East, and about Boston in particular, but has also tried a new compound for *filling*, which, I am sorry to learn, proved too *slippery* for *sticking*.

This Boston harbor,
With its oily Chowder,
In Western stomachs,
Will kick up a rumpus.

The fact is, it will not do for ballast; and I am told that even old and well-tried Yankee bottoms, on this occasion, had to throw it overboard.

If your space will permit, I should be pleased to give your Western readers (who were not so fortunate as to be at this meeting, and who may not have seen all the rich and interesting accounts of the same), a short account of the *extra* doings of this Convention, as well as a few remarks, which their published Minutes suggest.

From the *reports*, official as well as unofficial, which have have come to my knowledge, the famous scene at Nahant will long be cherished as the most thrilling of any in which the profession was ever engaged.

It appears that, after the excessive stirring up in the harbor, this celebrated outpost of Massachusetts—this famous

*watering* place, was taken by storm. It must be borne in mind that this is the retreat for the gentry of Boston. Here, during the heat of summer, the *elite* retire from the dust and hubbub of the city, and hence, here is the place to see a miniature world in fashion. Here you may see the lawyer, with his long, wise face on, who, as he promenades up and down, digests Kent, Blackstone, and his Codfish all together. Here you may see the doctor let loose from his patients; with his smiling face, you would think he had cast aside all care, and had got out of the reach of malaria. That man with the quick, business air is a merchant. The cool, bracing air "off Nahant" makes him think of his fall trade; and he can scarce wait time's rapid pace for business. You must see, for I know you can not help it, the stately mammas and simpering Misses, the belles that are *bells* indeed; for in circumference they would outmeasure those of our churches, and for ringing, they require no sexton with his long rope and see-saw, double pull, to keep them going. Here you may see belles that never grow older than twenty, and the blooming ones of "sweet sixteen"—*such luscious, enticing creatures!* No wonder Adam fell, and the younger ones have been falling ever since. Here, too, you may see, at times, a few live lords from Old England; and, as a matter of course, and which should not, at the present, be forgotten, a few of the Army and Navy; for on this peninsular outpost of the nation, some of her most heroic defenders are always stationed. These can always be recognized by their erect, precise, West Point air, as well as by the exhibition of a few *extra gilt* brass buttons.

It appears that the Convention arrived just when the guard had been "*drawn off*" for dinner. In a time of such general peace, no danger was apprehended. Hence, the "martial strains of music," with the soul-sickening "cries of the wounded," more dreadful than at the "battle of Prague," with the banner streaming in the breeze, showing in large letters, "Victory or death," "*a long pull, a strong pull*, and a pull all together," struck consternation into the unprepared garrison.

Veteran soldiers, however unprepared for defense they may be, are always (as on this occasion) sure to give battle. Hence, by the time the Convention had drawn up in front of the garrison (which is now used *somewhat* as a hotel), the besieged, under the admirable tactics of the Commanding General, had prepared for defense, so that all the doors and windows were guarded by the most *gallant* defenders. Unfortunately, the muskets and other arms had been left without; and in the hurry and excitement of the moment, such missiles as were most convenient were laid hold of for weapons. Some had a leg of mutton; some, roasted pigs; some, hams; and a great many had bottles (many of which were yet full of wine). The table *groaned* no more with the luxuries of life, so that even Gen. "Phœnix" had to grasp his fork, and load it with a "large potato," from "*which he had partially removed the cuticle.*" "To your defenses! O! ye Nahanters, for your wives and your little ones!" appeared to be the war-cry of the besieged. The Convention, in wonder and amazement at this unlooked-for reception, stood aghast; and in profound silence "*showed their teeth,*" when all at once, loud above the screams of the women and children, was heard the voice of Gen. "Phœnix," in thrilling tones, giving orders of battle to Major Hiram (of Tyre), and "Col. *Doelittle*, of Androscoggin."

The Colonel could be heard swearing by "*Jerusalem;*" but soon was heard, in notes of thunder from the General, "make ready, take aim, FIRE," when instantly the air was filled with a discharge of pigs, chickens, hams, bottles, and potatoes. Owing, however, to the distance, or the want of sufficient powder, only one ball took effect, and this hit Dr. Inventibus on the head, "when he squalled out he was dead;" but it was found that the "*ball*" was a potato, "*with the cuticle partially removed,*" the inside of which was, at first, taken for the poor man's brains. The Convention returned the fire with a discharge of cigars, then reloaded, each with a "large quid of tobacco," when "Dr. Tushmaker," as a final charge and coup d'etat, ordered each "to draw," when "*in an instant* every one was armed with a *brilliant* turnscrew;" the Doctor next gave the command to "*hawl*," when the redoubted General "Phœnix" *shouted* at the top of his voice, "Hold, for God's sake," "*the ladies with a wild cry of horror fled*, and a general surrender at once took place, Gen. Phœnix agreeing to pay all the expenses, including the bill for liquor, which, it is said, he since refuses to do, as so much was thrown away at the first discharge of his troops. The results of this battle show how "*wonderful are the improvements of science.*" The profession should have the thanks of the nation, and the ladies in particular, for having inaugurated a new system of military tactics. Dr. Inventibus recovered, and, after tanning the remaining "*cuticle* of the potato," has added a leather vest to his wardrobe.

After performing a few tricks of legerdemain on "snaggle tooth Bill," and giving the natives a sample of exquisite music for the benefit of the frightened children and nervous women, the convention, at 5 P. M., left the scene of *disaster*, and, to the tune of "*Pull, Brothers, pull*," moved off to Boston.

In relation to the *regular* proceedings of the convention, we are struck with the spirit and good feeling which appears to have characterized the discussion. It is to be hoped, however, that, as the profession get more used to such discussions, there will be more conciseness, and less wandering from the simple question. A few prompt, clear-headed men are always needed, in every deliberative assembly, to direct and keep the discussions in the right channel. It might, for instance, be asked with propriety, what is the difference between constituting a few members of the convention a committee for a certain purpose, or making the officers that committee? It is mere Tweedledee and Tweedledum.

The first regular subject for discussion was a very important one, embracing the very foundation-stone of dental science, "*What are the best means for securing a healthy denture?*" Judging from the discussion, the question must have been, "What are the best means to *preserve* a healthy denture?" There is certainly a great difference in the meaning of *preserve* and *secure.* We judge the intelligent agriculturist would discuss this question in a very different manner. Judging from the papers they publish, we would suppose that science is doing much to *secure* good crops. Even poor and worn-out soil is fed with the right food to secure this. Science points out the proper food for each kind of soil, and also for the crop to be raised. We have varied *soils* in the human system, all of which have peculiar constitutional characteristics, and each of them pointing out the kind of nutrition required to secure certain developments. We believe, with the King of Prussia and Prof. Townsend, that the seed and soil both should be good, to secure a good denture; yet as in this country we can not manage this affair to always gain this result, it is right and proper that we should call in the aid of science to remedy the evil; and, like the farmer, enrich the soil and secure a good crop of teeth. We believe that it is generally the duty of the mother to nurse her offspring; yet if her constitution is feeble, and she can not give the proper nutrition to her child, humanity would say, seek a substitute.

The paper on Miss *Sal-Eratus*, and her cousin, Miss Cream of Tartar, with her second cousin, Miss Carbonate of Soda, appears to have almost entirely changed the discussion from the direction it should have taken. It appears, however, to have been carried on with much ability, and to have elicited much sound argument; yet the fact that those residing on the sea shore suffer more from dental caries than those far in the interior, wants confirmation. Besides, if the theory advanced as its solution be correct, should we not expect to see the rugged limestone rocks on the sea shore crumbling beneath the touch of this liberated hydrochloric acid? Perhaps, however, nature somehow furnishes an *alkali* to fight this *acid*, and preserve these rocks. Does the sea-girt *fortress* of Nahant stand on a limestone foundation, or not?

If we had time, it would be pleasant to run over the whole of the discussions, and note, with approbation, the remarks of several gentlemen on filling teeth, the treatment of alveolar abscess, the annealing and purifying of gold, and the tempering of instruments, etc., etc.

The fact is, we have so much enjoyed the reading of what was said and done at Boston, that, if spared until your next meeting, we will try and get off our old fogy coat, and be a looker-on in the Queen City, next August. DENTATUS.

